# LIVING WITH MULTIPLE MYELOMA: SELF-MANAGEMENT STRATEGIES

**DOI:** 10.1093/geroni/igz038.3379

**Published:** 2019-11-08

**Authors:** Matthew R LeBlanc, Thomas W LeBlanc, Bryant L Ashley, Kathryn I Pollak, Donald E Bailey, Sophia K Smith

**Affiliations:** 1 Duke University School of Nursing, Durham, North Carolina, United States; 2 University of North Carolina Chapel Hill School of Nursing, Chapel Hill, North Carolina, United States

## Abstract

Multiple myeloma (myeloma), is an incurable cancer of the plasma cells that affects many older adults. Over 30,000 new diagnoses and over 12,000 deaths are attributed to myeloma annually in the United States, where the median age of diagnosis is 69 years old. Dramatic improvements in survival over the past fifteen years have transformed myeloma into a chronic disease for many. The disease and its toxic, ongoing treatment lead to significant challenges for patients. In this study we explore the self-management strategies patients use to address the challenges of living with myeloma through semi-structured one-on-one interviews with myeloma patients and clinicians. Fifteen myeloma patients and ten myeloma clinicians were interviewed between September 2017 and September 2018. Self-management strategies emerged in five major categories; managing uncertainty, finding emotional strength, seeking support, medication management, and activity management. The care of MM patients has made great strides as new and more effective treatments have extended survival for many patients. Effective self-management strategies are critical in addressing the challenges of this increasingly chronic disease. Our study explores the ways myeloma patients address the many challenges they face due to their disease and its’ treatment. Findings from this study could inform the development of interventions to optimize and support patients living with myeloma self-management.

